# First National Report on Aminotransaminases’ Percentiles in Children of the Middle East and North Africa (MENA): the CASPIAN-III Study

**DOI:** 10.5812/hepatmon.7711

**Published:** 2012-11-30

**Authors:** Roya Kelishadi, Seyed-Hossein Abtahi, Mostafa Qorbani, Ramin Heshmat, Mohammad Esmaeil Motlagh, Mahnaz Taslimi, Tahereh Aminaee, Gelayol Ardalan, Parinaz Poursafa, Payam Moin

**Affiliations:** 1 Department of Pediatrics, Child Growth and Development Research Center, Faculty of Medicine, Isfahan University of Medical Sciences, Isfahan, IR Iran; 2 Medical Students’ Research Center, Isfahan University of Medical Sciences, Isfahan, IR Iran; 3 Department of Epidemiology and Biostatistics, Tehran University of Medical Sciences, Tehran, IR Iran; 4 Department of Epidemiology, Endocrinology and Metabolism Research Center, Tehran University of Medical Sciences, Tehran, IR Iran; 5 Department of Pediatrics, Ahvaz Jundishapur University of Medical Sciences, Ahvaz, IR Iran; 6 Bureau of Population, Family and School Health, Ministry of Health and Medical Education, Tehran, IR Iran; 7 Bureau of Health and Fitness, Ministry of Education and Training, Tehran, IR Iran

**Keywords:** Aminotransferase, Child, Iran

## Abstract

**Background:**

By the current global obesogenic environment, non-alcoholic fatty liver disease is becoming an important health problem in the pediatric age group.

**Objectives:**

This study aimed to determine the first age-and gender-specific percentiles and upper limit normal limit (ULN) of alanine aminotransaminase (ALT) and aspartate aminotransaminase (AST) among a nationally-representative sample of children and adolescents in the Middle East and North Africa (MENA). The second objective was to determine the linear association of obesity indexes and age with serum ALT and AST levels.

**Patients and Methods:**

This nationwide study was conducted among a representative sample of 4078 students aged 10-18 years, who were selected by multistage random cluster sampling from 27 provinces of Iran. ALT and AST were measured on fresh sera. Body mass index (BMI) was calculated as an index of generalized obesity, and waist- to- height ratio (WHtR) as an index of abdominal obesity. The age- and gender-specific percentiles of ALT and AST were constructed, and the 95th percentile of each enzyme was considered as the ULN. Gender-specific linear regression analysis was employed to examine the association of BMI or WHtR with the levels of ALT and AST.

**Results:**

Data of ALT and AST were available for 4078 (2038 girls) and 4150 (2061 girls),respectively. Participants had a mean (SD) age of 14.71 (2.41).The ULN of ALT for boys, girls,and the total individuals were 36.00; 38.00; and, 37.00 U/L, respectively. In both genders, ALT and AST had linear association with age. The association with BMI was significant for ALT in both genders and for AST only in boys, the association of ALT with WHtR was significant in both genders; the corresponding figures were not significant for AST.

**Conclusions:**

The findings of the current study confirmed the current ULN value of 40 U/L commonly used for the pediatric age group. The linear association of indexes for generalized and abdominal obesity with ALT underscores the importance of timely prevention and control of childhood obesity.

## 1. Background

By the increasing trend of childhood obesity and other environmental factors, non-alcoholic fatty liver disease (NAFLD) is no more limited to adult population, and is becoming an important health problem in the pediatric age group, as well (1, 2). The main underlying cause is excess weight, but it may also occur in normal-weight children and adolescents (3-5). Aminotransaminases including alanine aminotransaminase (ALT) and aspartate aminotransaminase (AST) are commonly considered as liver function tests. However, since in addition to liver, AST is produced in other tissues as heart, muscle, kidney and brain, ALT has been generally accepted as a better predictor of liver injury. Currently in clinical setting, ALT level of 40 U/L is often considered as the upper limit of normal (ULN) in children and adults (6-8). Some studiesindicated this ALT cutoff level as moderate sensitivity for the pediatric age group; it is proposed that lower levels of ALT may be considered for children and adolescents rather than for adults (9-11). The limited studies that established the normal values of liver function tests in the pediatric age group proposed that the liver enzymes levels are different according to age and gender (12-14).

There is a growing body of evidence about the association of NAFLD with metabolic syndrome, as well as the common pathophysiological features of these two disorders (5, 14-17). Because of the considerably high prevalence of childhood obesity at global level, an escalating trend in the incidence of pediatric NAFLD can be expected in near future. Thus, it is important to determine the reference values and the ULN of liver function tests to facilitate the diagnosis of NAFLD in young age. Of special concern in this regard, are the children and adolescents living in the Middle East and North Africa (MENA), facing a rapid epidemiologic transition (18, 19), along with an ethnic predisposition to metabolic syndrome (20). To the best of our knowledge, only local studies have determined these reference values in the pediatric age group in non-Western countries (4, 14).

## 2. Objectives

The current study aimed to determine the first ageand gender-specific percentiles and ULN of ALT and AST among a nationally-representative sample of children and adolescents in MENA. The second objective was to determine the linear association of obesity indexes and age with serum ALT and AST levels.

## 3. Patients and Methods

This study was conducted as a part of the national survey of school student high risk behaviors” (2009-2010) as the third survey of the school-based surveillance system entitled Childhood and Adolescence Surveillance and Prevention of Adult Non communicable disease (CASPIAN-III) *. The methodology details including data collection and sampling framework of this study have been already explained (21), and here presented in brief. The whole survey included 5528 students (2726 girls, 69.37% urban, mean age 14.7 + -2.4 years) who were recruited by multistage random cluster sampling from urban and rural areas of 27 provincial counties in Iran. From the total sample under study, ALT and AST data were available for 4078 students, which were reported here. Ethics committees and other relevant national regulatory organizations approved the study. The team obtained written informed consent from parents and oral assent from children and adolescents. A team consisting of physicians, nurses, and expert health care professionals trained for the survey conducted the field examinations. Participants were selected by multistage-random cluster sampling from elementary, intermediate, and high school students of urban and rural areas of different counties. Those students with history of any acute or chronic diseases and any medication use were not included in the study.

A trained team of health professionals conducted the physical examination under standard protocols by using calibrated instruments. For blood sampling, students were invited to the nearest health center to the school. They were instructed to fast for 12 hours before the screening; compliance with fasting was determined by interview in the morning of examination. The blood samples were centrifuged for 10 minutes at 3000 rpm within 30 minutes of venipuncture. In each county, the biochemical analysis was performed in the Central Provincial laboratory which met the standards of the National Reference laboratory, a collaborating center of the World Health Organization in Tehran. The fresh sera were tested for ALT and AST levels with Pars Azmoon reagents kit (Tehran, Iran). Body mass index (BMI) was calculated as weight (Kg) divided by height squared (m^2^). Waist- to- height ratio (WHtR), computed by dividing waist circumference (cm) to height (cm), was considered as an index of abdominal obesity (22).

### 3.1. Statistical Analysis

Data are presented as mean ± standard deviation (SD). The age- and gender-specific 5th, 25th, 50th, 75th and 95thpercentiles of ALT and AST were constructed from the raw data. The 95th percentile of each enzyme was considered as the ULN. Gender-specific linear regression analysis was employed to examine the association of BMI or WHtR with the levels of ALT and AST. The results of gender-specific linear regression analyses are presented as R square and P values. SPSS software, version 16.0 (SPSS Inc., Chicago, Illinois, USA) was employed to conduct the data analysis. The significance level was P < 0.05.

## 4. Results

**Figure 1:**
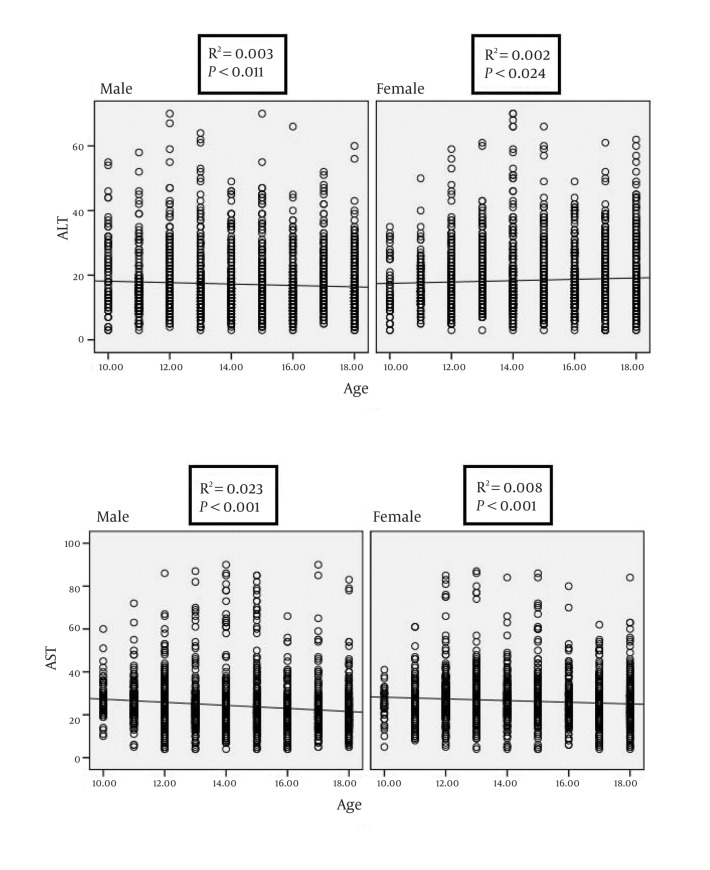
Gender-specific linear regression analysis on the association of alanine- and aspartate aminotransaminase levels with age: CASPIAN-III Study

**Figure 2:**
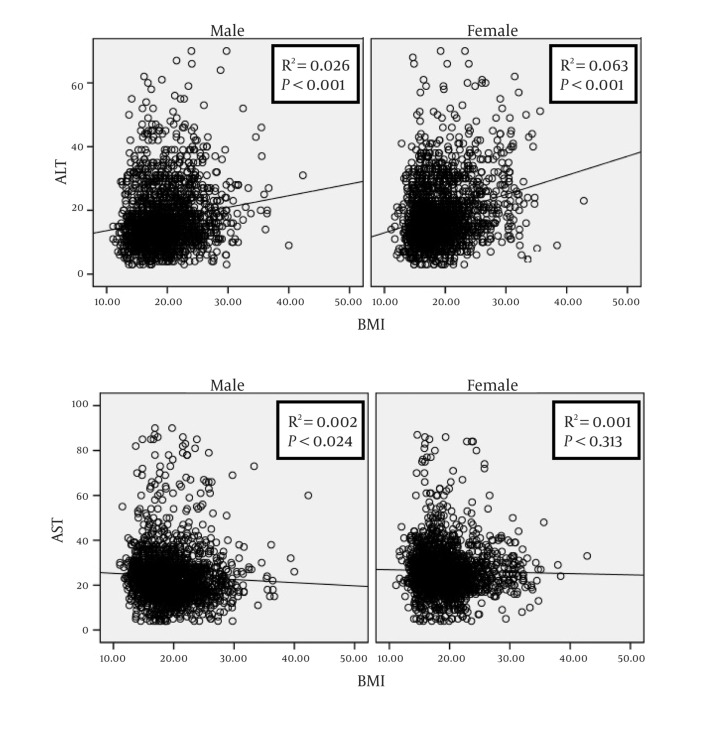
Gender-specific linear regression analysis on the association of alanine- and aspartate aminotransaminase levels with body mass index

**Figure 3:**
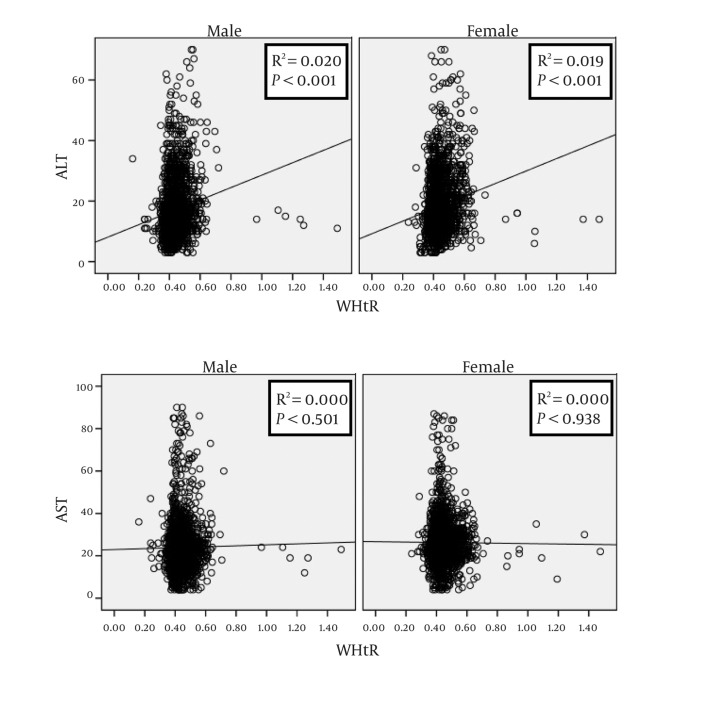
Gender-specific linear regression analysis on the association of alanine- and aspartate aminotransaminase levels with waist-to-height ratio

From the total sample under study, ALT and AST data were available for 4078 (2038 girls) and 4150 (2061 girls), respectively. These participants had a mean (SD) age of 14.71 (2.41) years, the corresponding figures for BMI and WHtR were 19.40 (4.14) Kg/m^2^ and 0.44(0.07), respectively. The age- and gender-specific 5th, 25th, 50th, 75th and 95th percentiles of ALT are presented in (Table 1), and the corresponding figures for AST are presented in (Table 2). The ULN (95th percentile) of ALT for boys, girls, and the total individuals were 36.00; 38.00; and, 37.00 U/L, respectively. By increasing age, from 10 to 18 years, the mean ± SD of ALT and AST concentrations ranged respectively from 20.90 ± 11.51 to 16.16±7.97U/L and 27.39 ± 7.76 to 21.51±9.49 U/L among boys; and from 16.11 ± 8.60 to 18.73 ± 10.32 U/L, and 25.31 ± 6.820 to 24.77 ± 9.46U/L among girls. Figures 1,2,3 illustrate the results of gender-specific linear regression analyses as well as the related linear R square and P values. Figure 1 shows the association of ALT and AST with age. In this analysis, the ALT- or AST-age association reached the statistical significance level in both genders. The association of ALT or AST with BMI was significant for ALT in both genders and for AST only in boys (Figure 2). As presented in Figure 3, the association of ALT with WHtR was significant in both genders; the corresponding figures were not significant for AST.

**Table 1:** Reference Values for Alanine Aminotransaminase According to age and Gender: CASPIAN-III Study

	**Age, y**	**No.**	**Mean ± SD**	**5^th^, %**	**25^th^, %**	**50^th^, %**	**75^th^, %**	**95^th^, %**
**Boys**	10	78	20.90 ± 11.518	4.0	12.75	18.00	29.00	44.10
	11	156	16.92 ± 9.620	5.00	10.00	15.00	19.00	39.00
	12	274	17.35 ± 9.907	7.00	11.00	14.00	21.00	35.75
	13	230	17.36 ± 10.726	6.00	11.00	15.00	20.00	39.45
	14	208	17.42 ± 9.569	6.00	11.00	15.00	21.00	37.00
	15	284	17.19 ± 9.447	6.00	11.00	15.00	20.00	38.50
	16	219	16.04 ± 8.780	6.00	10.00	14.00	19.00	34.00
	17	240	17.79± 8.653	8.05	12.00	16.00	21.00	36.00
	18	351	16.16 ± 7.975	6.00	11.00	15.00	19.00	31.00
	Total	2040	17.14 ± 9.415	6.00	11.00	15.00	20.75	36.00
**Girls**	10	54	16.11 ± 8.604	4.50	9.75	13.50	25.00	32.25
	11	114	17.41 ± 7.272	7.00	12.00	16.00	22.00	31.25
	12	288	17.02 ± 8.752	7.00	11.00	15.00	20.00	33.00
	13	247	19.12 ± 8.994	8.00	13.00	17.00	23.00	36.60
	14	220	19.74 ± 12.916	7.00	11.00	16.00	23.00	50.00
	15	248	18.42 ± 10.208	7.00	12.00	16.00	22.00	38.00
	16	215	18.55 ± 9.204	7.00	12.00	16.00	23.00	39.20
	17	282	19.04 ± 9.593	7.00	12.00	17.00	25.00	37.85
	18	370	18.73 ± 10.320	7.00	11.00	15.00	23.00	40.45
	Total	2038	18.49 ± 9.883	7.00	12.00	16.00	23.00	38.00
**Total**		4078	17.82 ± 9.673	7.00	11.00	16.00	22.00	37.00

**Table 2:** Reference Values for Aspartateaminotransaminase According to age and Gender: CASPIAN-III Study

****	**Age, y**	**No.**	**Mean ± SD**	**5^th^, %**	**25^th^, %**	**50^th^, %**	**75^th^,%**	**95^th^, %**
**Boys**	10	80	27.39 ± 7.768	14.25	23.00	26.00	29.75	41.90
	11	159	26.33 ± 9.275	14.00	20.00	25.00	30.00	42.00
	12	282	25.65 ± 10.692	10.00	20.00	24.00	30.00	42.85
	13	231	24.28 ± 12.224	9.20	17.00	23.00	27.00	48.60
	14	220	25.43 ± 14.812	10.00	18.00	22.00	28.00	43.00
	15	292	24.96 ± 14.424	10.00	17.00	22.00	28.00	42.05
	16	220	22.05 ± 9.308	8.05	16.25	21.00	26.00	38.95
	17	243	21.77± 10.867	10.00	15.00	20.00	25.00	40.00
	18	362	21.51 ± 9.495	10.00	16.00	20.00	20.00	37.00
	Total	2089	23.95 ± 11.603	10.00	17.00	22.00	28.00	43.00
**Girls**	10	55	25.31 ± 6.820	12.40	23.00	25.00	30.00	37.20
	11	122	25.64 ± 10.091	10.00	19.75	25.00	31.00	45.55
	12	292	28.07 ± 11.313	14.00	21.00	27.00	32.00	45.40
	13	251	27.96 ± 11.994	13.00	21.00	26.00	32.00	45.40
	14	211	26.84 ± 10.303	12.00	21.00	27.00	31.00	44.40
	15	247	27.53 ± 12.086	14.00	21.00	25.00	31.00	44.60
	16	219	25.44 ± 9.719	14.00	19.00	24.00	30.00	43.00
	17	285	24.94 ± 9.200	11.00	19.00	24.00	29.00	41.40
	18	379	24.77 ± 9.469	10.00	19.00	24.00	29.00	41.00
	Total	2061	26.33 ± 10.532	12.00	20.00	25.00	30.00	43.00
**Total**		4150	25.13 ± 11.147	11.00	19.00	24.00	29.00	43.00

## 5. Discussion

Data and percentiles of ALT and AST for 10-18-year-old Iranian children and adolescents were presented. To the best of the researchers’ knowledge, it was the first study reporting data of a nationally-representative sample from the pediatric age group in a non-Western population. Recruitment of nationally representative sample may allow to generalize the findings. The current study findings are consistent with the current ULN value of 40 U/L commonly used in clinical setting for the pediatric age group (6-8). A previous study in the urban area of the capital city of Iran revealed lower ULNs for ALT and AST (14). This difference may suggest that the children and adolescents living in the metropolitan Tehran may not be a representative sample of the whole country; moreover it may be because the sample size of the population studied in Tehran was less than one-fourth of the current nationwide study. Finding the serum ALT ULN of 40 U/L in children and adolescents aged 10 to 18 years is in line with the findings of a birth cohort showing the same cutoff for pre-school aged children (23). In the current study, ALT and AST levels were significantly associated with age. This finding is consistent with some previous studies in children (23) and adults (24); however some studies did not confirm such association in adults (25).

In the current study, the mean and median values of ALT and AST were higher in boys than in girls. This finding is consistent with some previous studies in Iranian children and adolescents (5, 14, 17, 26). In a study in Taiwan, the 2.5th-97.5th percentiles of ALT in children are reported 8-38 IU/L with significantly higher levels in boys than in girls (13). Likewise, in Australian adolescents both enzyme values were higher in boys than in girls (12). It can be suggested that irrespective of ethnicity and genetic background, ALT is higher in male than in female children and adolescents.

Although in the pediatric age group, increased ALT has different etiologies as inherited metabolic disorders, malnutrition, infections, and drug toxicity; in most cases, it is related to excess weight, and the relative changes in BMI may be related to the onset of fatty liver (27, 28). Consistent with previous studies in various age groups (8, 14, 24, 25), significant linear association was found between increasing BMI and serum ALT level. Accumulating body of evidence supports the health hazards and metabolic consequences of central fat deposition in the pediatric population (29-32). The current study finding on the linear association of WHtR, as an index for abdominal obesity, with ALT serves as a confirmatory evidence on the consequences of central obesity even in children and adolescents. Moreover, abdominal obesity is a core component of metabolic syndrome, and on the other hand, the interrelation of metabolic syndrome and NAFLD among children is well-documented (5, 29-32). These associations propose the common pathophysiological features of metabolic syndrome and fat deposition in liver from childhood. Study limitations and strengths: Potential limitations of this project in such a large scale was its cross-sectional nature and lack of Tanner staging to determine the effect of pubertal stage on ALT and AST values. Also, as it is utilized in some similar large studies (24), specific blood tests e.g. hepatitis B surface antigen and anti- Hepatits C Virus (HCV) antibody were not performed, since the participants were (a) generally vaccinated for hepatitis B virus; and, (b) at an age group with low risk for HCV (33). Although, there remains the possibility that the presumed ‘healthy’ population contaminated with viral liver diseases, the number of such cases is likely to be very small. The strength of this study is presenting the unique data of a large number of nationally representative samples of pediatric population from the MENA region. This study provided data and percentiles of ALT and AST for a nationally representative sample of Iranian children, which may be generalizable to the MENA pediatric population. The current study findings confirmed the current ULN value of 40 U/L commonly used for the pediatric age group. The linear association of indexes for generalized and abdominal obesity with ALT underscores the importance of prevention and control of childhood obesity, notably in low- and middle-income countries facing an emerging epidemic of non-communicable diseases.
